# Relationship between the degree of antioxidant protection and the level of malondialdehyde in high-performance Polish Holstein-Friesian cows in peak of lactation

**DOI:** 10.1371/journal.pone.0193512

**Published:** 2018-03-01

**Authors:** Aleksandra Kapusta, Beata Kuczyńska, Kamila Puppel

**Affiliations:** Cattle Breeding Division, Animal Breeding & Production Department, Warsaw University of Life Sciences, Warsaw, Poland; University of Illinois, UNITED STATES

## Abstract

Lipid peroxidation can be described as a process under which free radicals attack carbon double bonds of omega-3 and omega-6 fatty acids. Whereas the end products of this process are reactive aldehydes, such as malondialdehyde (MDA). Lipid peroxidation leads to adverse changes in the nutritional value of milk; therefore, higher degree of antioxidant protection (DAP) ensures higher stability of dairy products by effecting their high antioxidative potential. Therefore, the purpose of this study was to demonstrate the relationship between the DAP and the level of MDA in high-performance Polish Holstein-Friesian cows in peak of lactation. Sixty-three Polish Holstein-Friesian cows were selected to the experiment according to: parity (all in the 2^nd^ lactation), phase of lactation (peak of lactation), cytological quality of milk (somatic cell count < 150 thousand/ml) and without diagnosed metabolic diseases. The data obtained were analyzed statistically by two–way ANOVA, and Tukey’s post-hoc test. After analysis of performance the cows were divided into 3 groups (twenty one cows in each group) based on milk yield and MDA concentration. The study revealed a significant effect of the lactation performance of cows on MDA levels in milk (P ≤ 0.01). The highest concentration of MDA (61.137 nM/mL) was shown in milk of cows yielding between 50.00 and 55.80 kg/day. The highest concentration of fat was found in milk in which the MDA level ranged from 48 to 86 nM/mL. Whereas, the inverse relationship was demonstrated in case of protein concentration. The highest level of protein was found in cows with MDA levels in the range of 18–28 nM/mL (P ≤ 0.01). The lowest MDA level (in the range of 18–28 nM/mL) was associated with the highest concentration of vitamin E, β-carotene, total antioxidant status (TAS) and DAP, measured in both milk and plasma. The obtained results show that lipid peroxidation leads to adverse changes in the nutritional value of milk; the highest DAP (7.89 x 10^−3^) was found in the cows with the lowest MDA concentration in milk.

## Introduction

Lipid peroxidation is a biological lipid oxidation, chain and free radical process, that results in the formation of peroxides of omega-3 and omega-6 fatty acids. This reaction consists of three stages: initiation, propagation, and termination; typical steps of free radical reactions and reinitiation. Products of lipid peroxidation include reactive aldehydes such as malondialdehyde and 4-hydroxynonenal [1]. Malondialdehyde is produced during the peroxidation of polyunsaturated fatty acids by the action of reactive oxygen species, as a result of the depletion of antioxidant systems. There are two forms of MDA: endogenous—from lipid peroxidation; and exogenous–delivered with food [2,3]. MDA modifies the physical structures of cell membranes and is indirectly involved in the synthesis of protein, DNA, and RNA. In addition, it has mutagenic and carcinogenic properties. MDA has been frequently used for many years as a biomarker for lipid peroxidation of omega-3 and omega-6 fatty acids because of its facile reaction with thiobarbituric acid [4,5]. Ayala et al. [5] reported, that the thiobarbituric acid (TBA) test is predicated upon the reactivity of TBA toward MDA to yield an red adduct; this test was first used to demonstrate autoxidative degradation of fats.

An antioxidant is a substance that, even in low concentrations compared to a substance that is susceptible to oxidation, significantly delays or prevents by minimizing oxidative damages in cells and biomolecules [1]. Antioxidants also reduce the risk of atherosclerosis, protect against ischemic heart disease, reduce the risk of cancer, slow down the course of Alzheimer's disease, and protect the body from environmental pollution [6]. Typical exogenous antioxidants supplied with food or in specially formulated supplements are: vitamins A, C, and E, and carotenoids (α- and β-carotene). Several forms of carotenoids may be found in cow’s milk: all-trans β-carotene, α-carotene, δ-carotene, zeaxanthin, and β-cryptoxanthin; with all-trans β-carotene occurring in the highest concentration—750 g/kg [7,8]. β-Carotene (provitamin A) reduces the risk of breast, lung, stomach, and uterine cancer [9], protects against myocardial infarction and stroke [10]. Rice-Evans et al. [11] have shown that carotenoids exhibit antioxidant properties and suppress free radicals by transferring free electrons, or by forming complexes with them.

One of the biologically-active compounds commonly known as vitamin E, is α-tocopherol. Tocopherol family consists of four representatives: α, β, δ, and Δ; all of them occur in the "D" form. D-α-tocopherol (RRR-α-tocopherol) is the most common and most active form, but not only is it antioxidant, similar properties are attributed to the tocopherol gamma form, as the only tocopherol to protect against nitrogen oxides [1]. Vitamin E (tocopherol) prevents: oxidation of LDL cholesterol fraction, heart disease, and cancer [7,12,13,14,15], it strengthens the immune system, inhibits the development of Alzheimer's disease, helps control blood sugar, protects the eye lens from damage caused by free radicals, and retards the development of Parkinson's disease [9]. Vitamin E is one of the most important antioxidants soluble in milk fat. It prevents lipid peroxidation by trapping a singlet oxygen, hydroxyl and superoxide radicals [7]. By entering the free radical reaction, a tocopherol is converted into a tocopherol radical; and ascorbic acid participates in its regeneration (by reducing it). Vitamin E and carotenoids protect fatty compounds against oxidation in the blood and within the cell membranes, and play a significant role in the communicative and defense functions of cells against free radicals [16,17].

Cholesterol (5-Cholesten-3β-ol) is an alcohol with the general formula C_27_H_45_OH and a sterol in the 17-carbon cyclopentane phenanthrene ring, linked to the C17 hydrocarbon chain with C10 and C13 hydroxyl groups, C3 hydroxyl group and one double bond between C5 and C6 atoms. Milk and dairy products are often regarded as examples of products with high levels of cholesterol having adverse effect on human health [18,19]. Tomaszewski [20] has shown that cholesterol levels in milk clearly increase with increased yield. In contact with air, cholesterol is autoxidized, and the rate of the formation of cholesterol oxidation products depends mainly on storage conditions. Cholesterol oxidation products (PUCHs) are highly toxic and highly reactive substances which initiate the free radical processes that may result in atherogenic and cancerous lesions. The most bioactive of these are: b-sitosterol (5-cholesten-24bethyl-3b-ol), campesterol (5-cholesten-24b-methyl-3b-ol), and stigmasterol (5,22-cholestadien-24-ethyl-3b-ol) [19,21]. Whole milk contains 20–30 mg PUCh/kg [22]. They are produced by non-enzymatic reactions such as ROS action, lipid peroxidation, and enzyme activity. Oxidative processes have a detrimental effect on milk and dairy products, by shortening their shelf-life and deteriorating their nutritional quality [23,24]. Havemose et al. [25] found that higher concentrations of antioxidants contributed to delaying or eliminating protein oxidation processes. Proper nutrition and supplementation provide antioxidant protection. In dairy products, the degree of antioxidant protection is calculated as the molar ratio between antioxidants (α-tocopherol and β-carotene) and the level of oxidants (cholesterol). Lipid peroxidation leads to adverse changes in the nutritional value of milk and its products; therefore, higher DAP ensures higher stability of dairy products by effecting their high antioxidative potential.

The main objective of the experiment described in this manuscript was to demonstrate the relationship between the degree of antioxidant protection and the level of malondialdehyde in high-performance PHF cows at the peak of lactation.

## Material and methods

The experiment was conducted at the experimental dairy farm of the Warsaw University of Life Sciences (WULS). All cows were handled in accordance with the regulations of the Polish Council on Animal Care, and the Warsaw University of Life Sciences Care Committee reviewed and approved the experiment and all procedures carried out in the study. At the time of sample collection the cows were under veterinary control and did not manifest any disease symptoms.

### Animals, treatment and sampling

The experiment was carried out at the experimental dairy farm of the WULS. The cows were kept in a free-stall dairy shed and fed a total mixed ration (TMR) diet (Table 1).

**Table 1:** Ingredient and chemical composition of the total mixed ration (TMR).

	TMR diet
**Ingredient [kg d**^**-1**^**]**	
Maize silage	24.0
Alfalfa silage	10.30
Corn silage	5.0
Soybean meal	1.80
Pasture ground chalk	0.10
VIT-RA BML- vitamin mix ^1^	0.16
Salt	0.05
Rapeseed meal	2.50
Magnesium oxide	0.05
**Chemical composition [g kg**^**-1**^ **DM]**	
Ash	63.0
Crude protein	95.0
Fat	45.5
Acid detergent fiber	230.0
Neutral detergent fiber	360.0
Ca	9
P	5
Total, kg of DM (offered)	21.2
Daily intake (kg)	20.8
UFL(unit of milk production.) balance (%)	+5.25%
PDIN (protein digested in the small intestine when rumen-fermentable nitrogen is limiting)	+ 3.43%
PDIE (protein digested in the small intestine when rumen-fermentable energy is limiting)	-2.78%

^1^VIT-RA BML (values per kg): 150 g Ca, 100 g P, 50 g Na, 40 g Mg, 9000mg Zn, 7000 mg Mn, 1000 mg Cu, 100mg J, 50mg Se, 1 200 000 IU vitamin A, 120 000 IU vitamin D_3_, 5 000 mg vitamin E, 93 mg vitamin K, 80 mg vitamin B_1_, 160 mg vitamin B_6_, 110 mg vitamin B_2_, 1 000 μg vitamin B_12_ (PPH VITRA, Kusowo, Poland).

Sixty-three Polish Holstein-Friesian cows were selected to the experiment according to: parity (all in the 2^nd^ lactation), phase of lactation (peak of lactation; at 70±18 days in milk), cytological quality of milk (somatic cell count < 150 thousand/ml) and without diagnosed metabolic diseases. The cows were milked daily at 05.30 and 17.30 h and milk yield was recorded at each milking. Combined milk from morning and evening milking were placed in sterile bottles, immediately chilled (up to 5°C), preserved with Mlekostat CC (Zekar Sp. z o.o., Brwinów, Poland) and submitted to the WULS Milk Testing Laboratory for chemical analysis.

Blood samples (10 mL) were taken from each cow (the day following the sampling of milk) by jugular venipuncture (by a veterinary doctor) into a heparinized tube, separated by centrifugation at room temperature (1,800×g, 15 min) andimmediately chilled (up to 5°C) Next, they were transported to the to the Cattle Breeding Division (Milk Testing Laboratory of WULS) for analysis. It should be noted that the analyzes were carried out on the same day, and samples were collected from all animals on the same day.

### Chemical analyses

Milk gross composition: fat, total protein and casein were determined by infrared spectrophotometry using Milko-Scan FT-120 analyzer (Foss Electric, Hillerod, Denmark).

Extraction of fat was performed according to Röse-Gottlieb procedure [26] at a room temperature. The determination of α-tocopherol (vitamin E) and β-carotene (BK) were established using an Agilent 1100 Series reverse phase high–performance liquid chromatograph (Agilent Technologies, Waldbronn, Germany) according to the methodology described by Puppel et al. [27]

The determination of MDA level were established using Tecan's NanoQuant Infinite M200 PRO (Tecan Austria GmbH, Grödig, Austria)analyzer at wavelength532nm. To 250 μL milk (in 2.0 ml Eppendorf cup), 25 μL 0.2% *2*,*6-bis(1*,*1-dimetyoetylo)-4-metylofenol* (BHT; in absolute ethanol) and 1 ml 5% trichloroacetic acid (aqueous; TCA; Sigma-Aldrich, Warsaw, Poland) was added and vortexed. After centrifugation at 14 000g for 10 minutes, 750 μL of clear supernatant was transferred to glass tube. A 500 μL 0.6% Thiobarbituric acid (aqueous; Sigma-Aldrich, Warsaw, Poland) was then added, mixed and incubated for 45 minutes in water bath in 90°C. MDA was reacted with TBA in the presence of BHT to minimize formation of artifacts. After cooled on ice and centrifugation at 4 000g for 5 minutes, 200μL of clear supernatant was transferred to the microplate. This method is based on the reaction of free MDA (present in the sample) with TBA to generate a MDA-TBA adduct. The MDA-TBA adduct can be easily quantified colorimetrically (OD = 532 nm). The commonly used TBA test for measuring MDA production has been criticized for its lack of specificity and low reproducibility [28]. Notwithstanding, measuring the end products of lipid peroxidation is one of the most widely accepted assays for oxidative damage.

The determination of Cholesterol level were established using Agilent 7890A GC and BP-5 column (Agilent Technologies, Waldbronn, Germany). Fatty acid methylation was performed according to the trans estrification method EN ISO 5509 [29]. After centrifugation at 3000 g for 3 minutes, 50 μL *N*,*O*-Bis(trimethylsilyl)trifluoroacetamide (BSTFA; Sigma-Aldrich, Warsaw, Poland), and 50 μL pirydyne (Sigma-Aldrich, Warsaw, Poland) were added, then mixed and incubated for 20 minutes in water bath in 75°C. After cooling, 500 μL n-heksane (Sigma-Aldrich, Warsaw, Poland) was added and transferred to the vials. The separation was performed at pre programmed temperature: 250°C for 4 min and 300°C for 15 min. Helium at a flow rate of 25 cm/sand constant pressure was used as the carrier gas.

Total antioxidant status were established by RANDOX application using an NanoQuant Infinietie M200Pro analyzer (Tecan Austria GmbH, Grödig, Austria). ABTS^®^ incubation with peroxidase (metmyoglobin) leads to the formation of the ABTS ^+^ radical cation. This substance is blue-green and can be detected at a wavelength of 600 nm. The antioxidants present in the sample decrease the formation of the blue-green color, in proportion to their concentration.

HX-FeIII–metmyoglobin, X-[Fe^IV^ = 0]–ferrylmyoglobin, ABTS^®^- 2,2-Azino-di[3ethylbenzthiazoline sulphonate] (RANDOX materials). U/L shoves concentration of TAS.

$${\mathrm{HX}‑\mathrm{Fe}}^{\mathrm{III}}+H_{2}O_{2}\to X‑[{\mathrm{Fe}}^{\mathrm{IV}}=0]+H_{2}O$$

$${\mathrm{ABTS}}^{®}+X‑[{\mathrm{Fe}}^{\mathrm{IV}}=0]\to {\mathrm{ABTS}}^{®+}+\mathrm{HX}‑{\mathrm{Fe}}^{\mathrm{III}}$$

DAP was calculated from the molar ratio between antioxidants and oxidants according to Puppel et al. [21] from Pizzoferrato et al. [30]

$$DAP=\frac{\sum_{i=1}^{n}{\mathrm{AC}}_{i}\;(n{}^{\circ}\mathrm{moles})}{\mathrm{OT}\;(n{}^{\circ}\mathrm{moles})}$$

Where: AC_i_ is the antioxidant compound, OT is the oxidation target (cholesterol) and *i* is the number of components.

### Statistical analysis

After preliminary analyses (analysis of cows performance and MDA concentration), the cows were divided into 3 groups (twenty one cows in each group) based on milk yield:

1. 36.80–39.90 kg/d
2. 40.00–49.20 kg/d
3. 50.00–55.80 kg/d

and MDA concentration in milk (twenty one cows in each group):

1. 18–28 nM/mL
2. 29–47 nM/mL
3. 48–86 nM/mL

The analyses were performed using SPSS 23.0 [31]. The data obtained were analyzed statistically by two–way ANOVA, and Tukey’s post-hoc test. Significant differences were present among the means at a 95.0% confidence level. Data were presented as least squares means with standard error of the mean.

The statistical model was:

Yijkl=μ+Ai+Bj+(Ai×Bj)+eijk

Where: Y_ijkl_ is the dependent variable; μ is the overall mean; A_i_ is the milk yield effect (i = 1, 2, 3,); B_j_ is the MDA level effect (j = 1, 2, 3,); (A_i_ x B_j_) is the interaction between milk yield and MDA level; e_ijk_ is the residual error.

## Results and discussion

Continuous genetic selection has improved the milk yield per cow in the past few decades. The increase in production should be viewed with concern because: the increase in milk yield has been accompanied by declining fertility, increasing locomotive and metabolic problems and declining longevity [32]. Ingvartsen et al [33]showed an unfavourable genetic correlation between milk yield and incidence of ketosis (0.26–0.65), ovariancyst (0.23–0.42), mastitis (0.15–0.68) and lameness (0.24–0.48).

**Fig 1:**
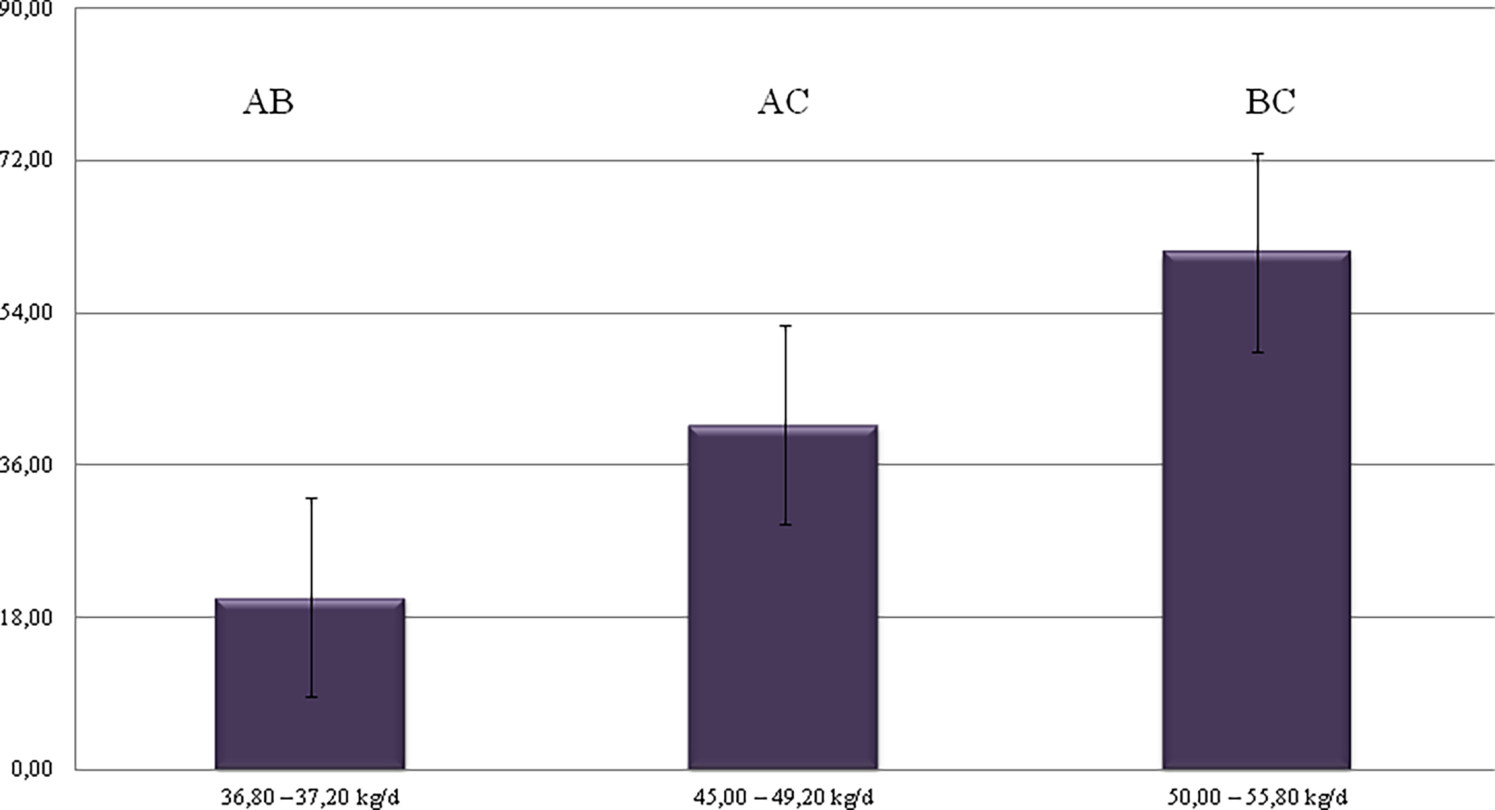
Changes in levels of MDA in milk, depending on cows performance.

Malondialdehyde, the lipid peroxidation end product is one of the most reliable and often used indexes of oxidative stress. The study demonstrated the high impact of the milking performance of cows on MDA levels in milk. The highest concentration of MDA—61.137 nM/mL–was shown in milk of cows from the 3rd group; yielding between 50.00 and 55.80 kg/d (Fig 1). This proves the negative effect of high milk yield of the cows on their milk quality. Cows producing large amounts of milk are more likely to be exposed to free radicals and therefore to free radical peroxidation processes which result in MDA production [34]. Castillo et al. [35] showed that after the peak of lactation, the metabolic status stabilizes (with mean values of 28.87 ± 5.33 μm/L for MDA) and MDA level decreases. Additionally Sharma et al [34], stated that dairy cows have more oxidative stress and low antioxidant defense during early lactation or just after parturition and it could by reason for their increased susceptibility to production diseases (e.g. mastitis, metritis, retention of fetal membranes etc.).

**Fig 2:**
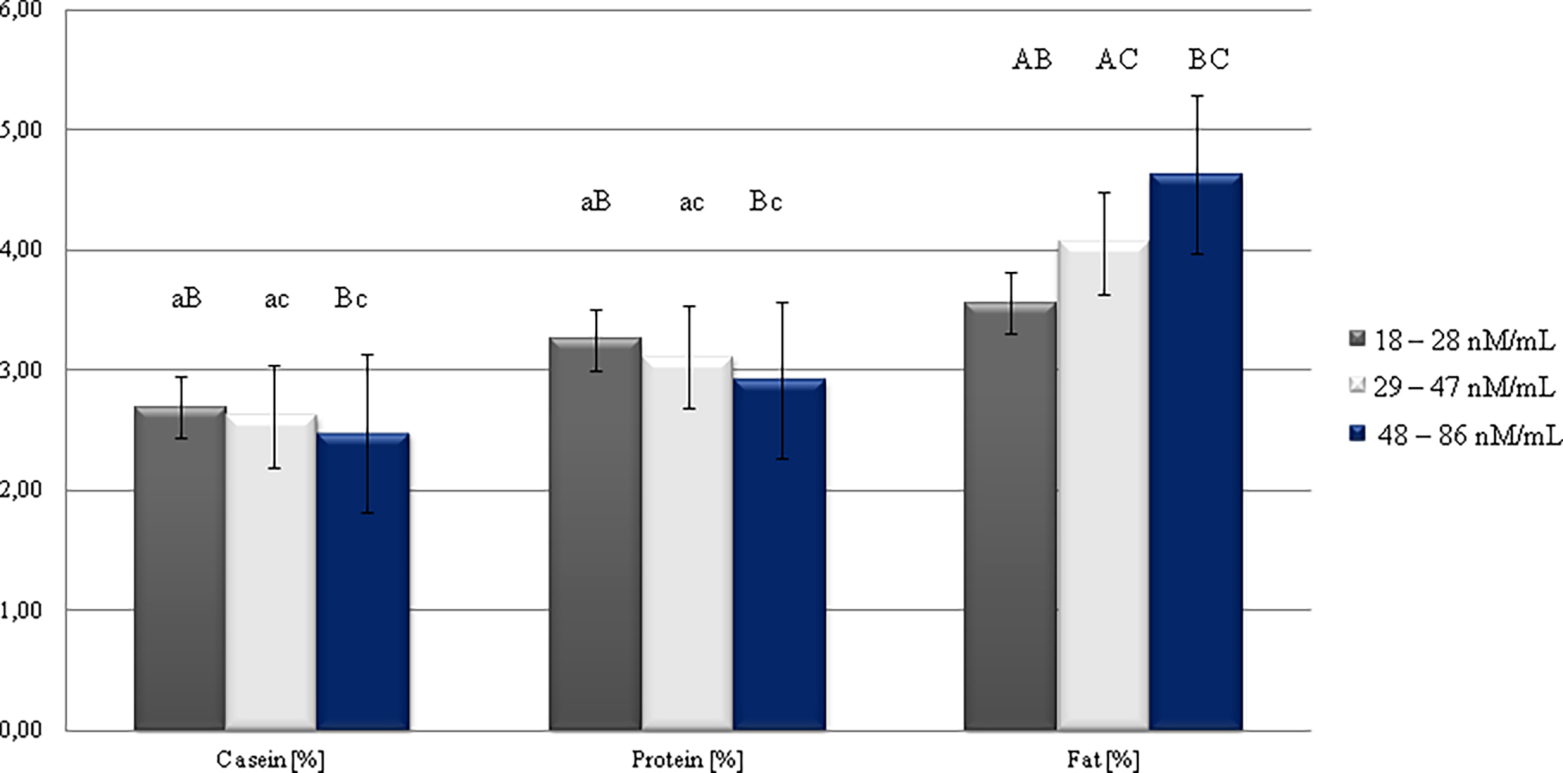
Changes in milk gross composition, depending on MDA concentration.

The study showed a significant connection between MDA concentrations and composition of cows' milk (Fig 2). The highest concentration of fat (4.63%) was found in milk in which the MDA level ranged from 48 to 86 nM/mL. Whereas, the inverse relationship was demonstrated in case of protein concentration. The highest level of this component (3.26%) was found in cows with MDA levels in the range of 18–28 nM/mL. On the other hand, Suriyasathaporn et al [36] reported, that results from Pearson’s correlation coefficients showed that MDA was significantly associated only with BMSCC (bulk tank SCC).

**Fig 3:**
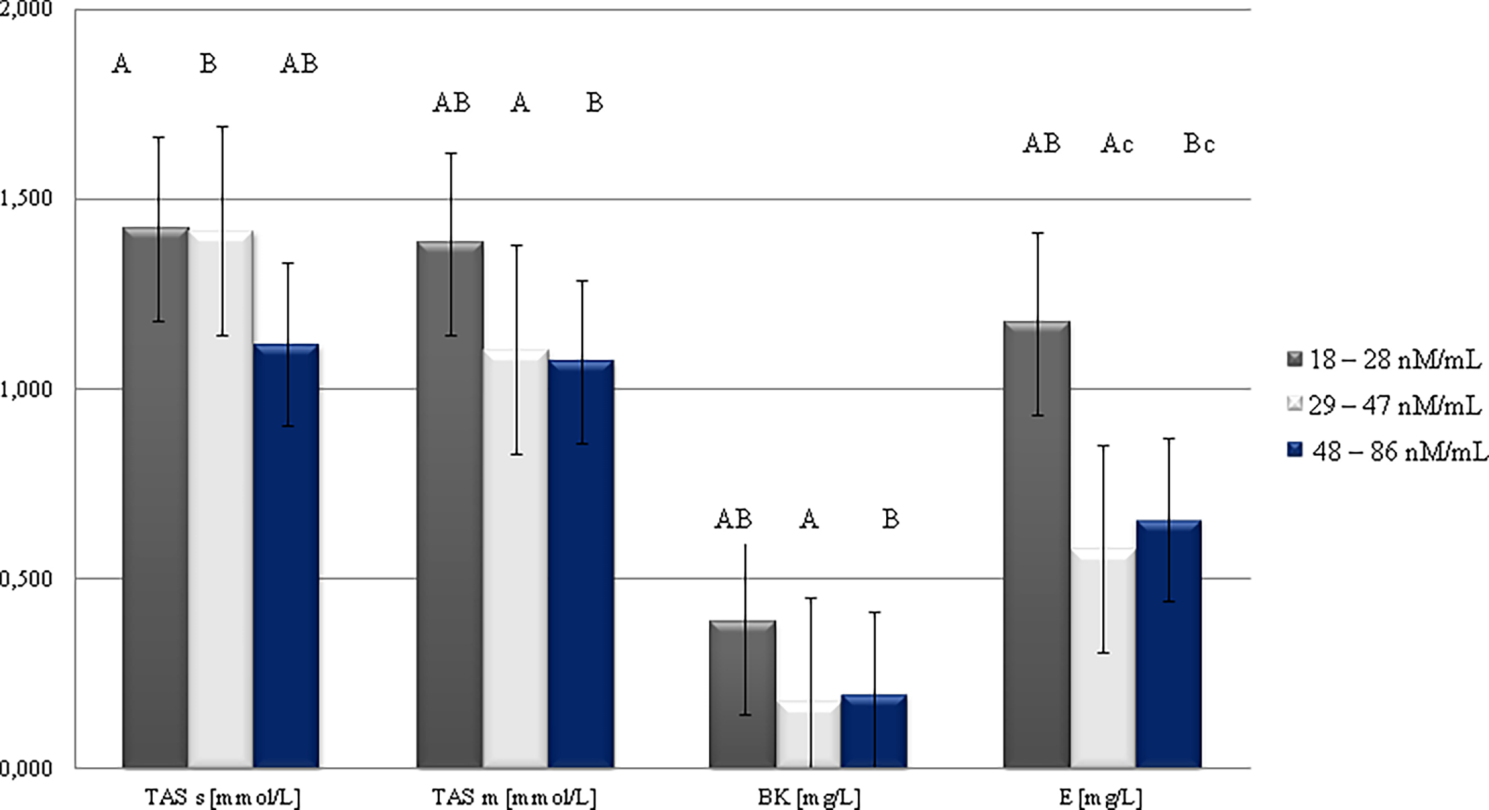
Changes in antioxidant capacity of milk depending on MDA concentration.

In healthy animals, the antioxidant system protects tissues from the attack of free radicals. There are three types of antioxidants: these of the first line of defense (superoxide dismutase, glutathione peroxidase, and celuroplasmin), these of the second line of defense (vitamin E, β-carotene) which remove newly created free radicals before they can trigger chain reactions, and these of the third line of defense (DNA repair enzymes, and methionine sulfoxide reductase) [1]. A kit for determination of the total level of antioxidants—TAS, enables the assessment of an integrated antioxidant system that covers all biological components which exhibit antioxidant activity. The obtained results show the high impact of MDA concentrations on the antioxidant potential of cow’s milk. The lowest MDA level was associated with the highest concentration of vitamin E (1.174 mg/L), β-carotene (0.387 mg/L) and TAS measured in both milk and plasma (1.384 and 1.423 mmol/L, respectively; Fig 3). Similar relationships has been shown by Pintea et al. [37] with regard to β-carotene. TAS and MDA levels are usually negatively correlated; i.e. once lipid peroxidation (MDA) increases, the antioxidant potential decreases. This correlation is especially visible in sick cows. Endometritis was associated with higher degree of oxidative stress (decreasing TAS and increasing MDA content) [2].

**Fig 4:**
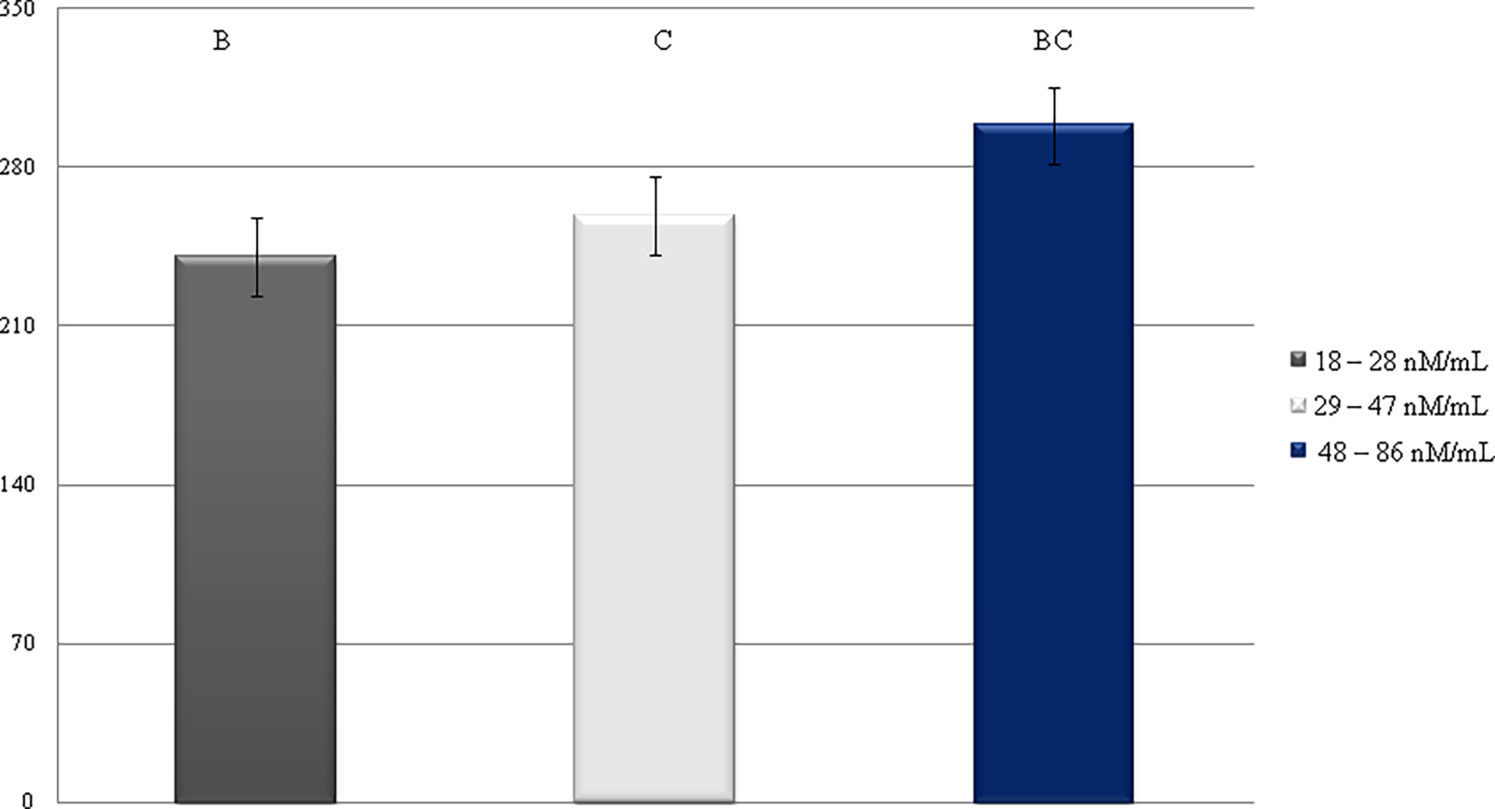
Changes in level of cholesterol milk depending on MDA concentration.

In contact with air, cholesterol undergoes autooxidation, and the rate of the formation of cholesterol oxidation products depends mainly on storage conditions [38]. Studies have shown the significant effect of MDA concentrations on cholesterol synthesis in milk fat (Fig 4). The highest level of cholesterol—298.43 mg/100 g of fat, was found in the cows with the highest concentration of MDA in milk; what is also confirmed by results of Pearson correlation (Table 2). Sparse information may be found in the available literature about the correlation between MDA and cholesterol levels. However other researches confirmed a correlation between cholesterol level and stage of lactation [39]. The highest content of cholesterol was found at the peak of lactation, which is in agreement with the highest concentration of MDA in milk determined in our study. Strzałkowska et al. [40] confirmed that cholesterol content of milk increased with the progress of lactation. Similar results were obtained by Son et al. [41].

**Table 2:** Pearson correlations between individual components.

	Milk yield	Casein	Protein	Fat	FY	MDA	TASs	TASm	BK	E	Cholesterol	DAP
**Milk yield**	1	NS	-0.351**	NS	0.254*	0.902**	-0.412**	-0.390**	-0.565**	-0.593**	0.556**	-0.689**
**Casein**	NS	1	0.942**	NS	NS	-0.252*	NS	NS	0.265*	NS	NS	NS
**Protein**	-0.351**	0.942**	1	NS	NS	-0.361**	NS	NS	0.322**	NS	NS	0.281*
**Fat**	NS	NS	NS	1	-0.845**	NS	NS	NS	NS	NS	NS	NS
**FY**	0.254*	NS	NS	-0.845**	1	NS	NS	NS	NS	NS	NS	NS
**MDA**	0.902**	-0.252*	-0.361**	NS	NS	1	-0.494**	-0.370**	-0.529**	-0.509**	0.695**	-0.645**
**TASs**	-0.412**	NS	NS	NS	NS	-0.494**	1	NS	0.391**	0.317*	-0.252*	0.386**
**TASm**	-0.390**	NS	NS	NS	NS	-0.370**	NS	1	NS	0.303*	-0.339**	0.370**
**BK**	-0.565**	0.265*	0.322**	NS	NS	-0.529**	0.391**	NS	1	0.521**	-0.403**	0.772**
**E**	-0.593**	NS	NS	NS	NS	-0.509**	0.317*	0.303*	0.521**	1	-0.343**	0.901**
**Cholesterol**	0.556**	NS	NS	NS	NS	0.695**	-0.252*	-0.339**	-0.403**	-0.343**	1	-0.589**
**DAP**	-0.689**	NS	0.281*	NS	NS	-0.645**	0.386**	0.370**	0.772**	0.901**	-0.589**	1

MDA- malondialdehyde; TASm- Total antioxidant status determined in milk; TASs- Total antioxidant status determined in serum; BK- β-carotene; E- α-tocopherol; DAP- degree of antioxidant protection; FY- fat yield

** The correlation significant at the 0.01 level (two-sided)

* The correlation significant at the 0.05 level (two-sided)

NS- not significant

**Fig 5:**
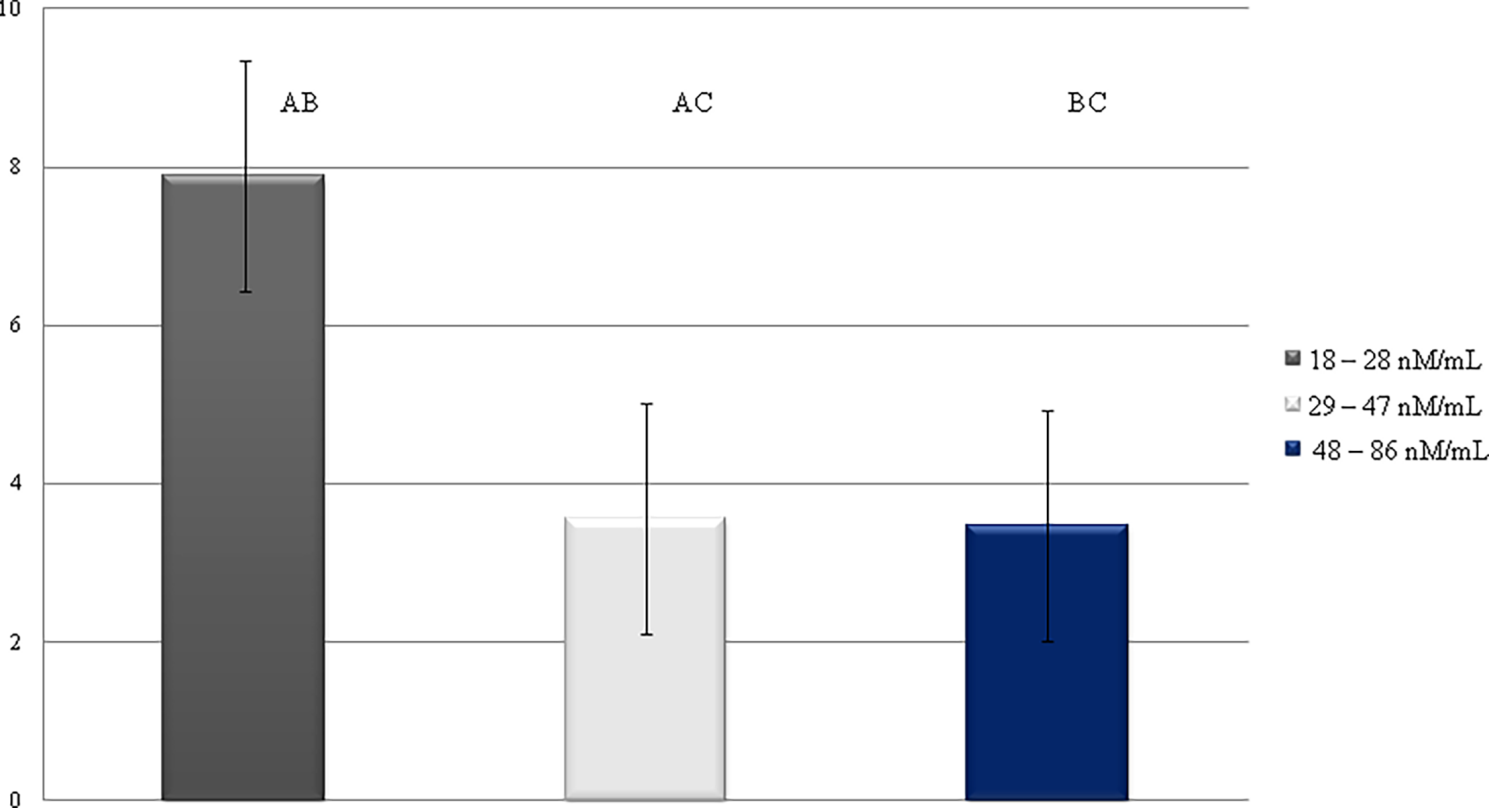
Changes in level of DAP, depending on MDA concentration.

Oxidative processes have a negative impact on milk and dairy products. In dairy products, the degree of antioxidant protection is calculated from the molar ratio between levels of antioxidants and oxidants. Lipid peroxidation caused by the oxidative stress, leads to adverse changes in the nutritional value of milk and milk products. Changes in the DAP are shown in Fig 5. The obtained result demonstrate the negative effect of MDA concentrations on DAP. The highest DAP (7.89 x 10−^3^) was found in the cows with the lowest MDA concentration in milk (Fig 5). Sharma et al [34], reported that in cattle oxidative stress and induction of the immune response increases nutrient requirements. Pizzoferrato et al. [30] proved that the highest content of DAP was based on grazing than when cut herbage was utilized indoors by animals. During grazing on pasture, more natural antioxidants from the forage are introduced into the ruminant’s body. They prevent lipid peroxidation and thus the formation of MDA, and cause DAP to increase. This confirms the negative relationship between MDA level and DAP found in our study.

The study showed a significant correlation between MDA concentrations and individual components (Table 2). As shown by study results, the high concentrations of MDA was associated with lowered level: TASs, TASm, BK, E and DAP.

## Conclusion

The results of this study enable formulating the following conclusions:

1. Milk yield of cows has a high impact on MDA levels in milk;
2. The level of cholesterol in milk is connected with the milk yield of cows;
3. As the concentration of MDA increases, the antioxidant capacity of milk decreases;

## Supporting information

S1 File The results obtained for individual cows. (PDF)
