# Supplementary material for: Relationship between the degree of antioxidant protection and the level of malondialdehyde in high-performance Polish Holstein-Friesian cows in peak of lactation
Source: PLoS One. 2018 Mar 1;13(3):e0193512. doi: 10.1371/journal.pone.0193512 (PMC5832249; doi:10.1371/journal.pone.0193512)
Supplement: S1 File — (PDF) [file pone.0193512.s001.pdf]

Table 1. Ingredient and chemical composition of the total mixed ration (TMR).

	**TMR diet**
**Ingredient [kg d^{-1}]**	
Maize silage	24.0
Alfalfa silage	10.30
Corn silage	5.0
Soybean meal	1.80
Pasture ground chalk	0.10
VIT-RA BML- vitamin mix [1]	0.16
Salt	0.05
Rapeseed meal	2.50
Magnesium oxide	0.05
**Chemical composition [g kg^{-1} DM]**	
Ash	63.0
Crude protein	95.0
Fat	45.5
Acid detergent fiber	230.0
Neutral detergent fiber	360.0
Ca	9
P	5
Total, kg of DM (offered)	21.2
Daily intake (kg)	20.8
UFL(unit of milk production.) balance (%)	+5.25%
PDIN (protein digested in the small intestine when rumen-fermentable nitrogen is limiting.)	+ 3.43%
PDIE (protein digested in the small intestine when rumen-fermentable energy is limiting.)	-2.78%

[1]VIT-RA BML (values per kg): 150 g Ca, 100 g P, 50 g Na, 40 g Mg, 9000mg Zn, 7000 mg Mn, 1000 mg Cu, 100mg J, 50mg Se, 1 200 000 IU vitamin A, 120 000 IU vitamin D_3, 5 000 mg vitamin E, 93 mg vitamin K, 80 mg vitamin B_1, 160 mg vitamin B_6, 110 mg vitamin B_2, 1 000 µg vitamin B_{12} (PPH VITRA, Kusowo, Poland).
